# Recurring Giant Conjunctival Cyst Effectively Treated With 20% Trichloroacetic Acid

**DOI:** 10.7759/cureus.74185

**Published:** 2024-11-21

**Authors:** José J López-Fontanet, Sebastian Ruiz, Angel G Torres, Marino Blasini, Ricardo Rodriguez Rosa

**Affiliations:** 1 Department of Ophthalmology, School of Medicine, Medical Sciences Campus, University of Puerto Rico, San Juan, PRI

**Keywords:** chemical cautery, giant conjunctival cyst, recurrent cyst treatment, strabismus surgery, trichloroacetic acid

## Abstract

Giant conjunctival cysts, though rare, can cause significant discomfort and functional impairment due to their size as well as their potential for rupture and recurrence. We report the case of a 51-year-old female who presented with a recurrent giant conjunctival cyst in her left eye, experiencing considerable discomfort and pain upon eye movement. The cyst, located in the left eye, had previously recurred after surgical excision, though visual acuity remained 20/20 bilaterally. Treatment involved aspiration of the cyst, injection of 20% trichloroacetic acid (TCA), and irrigation of the cyst cavity, leading to complete resolution with no recurrence or symptoms after eight months. This case suggests that 20% TCA is a viable and effective treatment option for recurrent giant conjunctival cysts, providing a less invasive approach with a reduced risk of recurrence.

## Introduction

Conjunctival cysts are benign, fluid-filled lesions that arise from the conjunctiva. The walls of these cysts are composed of layers of non-keratinized lining epithelium and connective tissue [1]. These cysts can result from trauma, inflammation, or congenital anomalies, and they are sometimes a complication of ocular surgery, such as strabismus surgery [2]. During strabismus surgery, manipulating the extraocular muscles can inadvertently trap conjunctival epithelial cells, which can, over time, lead to the formation of cysts [2,3]. Giant conjunctival cysts, although rare, can cause significant ocular discomfort, visual disturbances, and cosmetic concerns, impacting a patient’s quality of life. Symptoms may include foreign body sensation, ocular irritation, and pain, particularly when eye movement is restricted.

Traditional management includes surgical excision, which can carry recurrence risks and complications. Recently, less invasive approaches have been reported, such as intralesional injections of sclerosant agents like trichloroacetic acid (TCA) [4,5]. In this case report, we discuss a 51-year-old female patient with a giant conjunctival cyst as a complication of childhood strabismus surgery, where the cyst recurred after excision surgery. We assessed an alternative treatment with intralesional TCA injection, resulting in the successful resolution of the cyst and significant symptom relief, highlighting the efficacy of this novel treatment approach.

## Case presentation

A 51-year-old female patient presented to the ophthalmology pediatric and strabismus clinic, her chief complaint being a progressively enlarging mass in her left eye (OS) over the past 30 years. The patient had significantly developed ocular pain and discomfort when attempting leftward gaze, which was accompanied by constant headaches. Her medical history included hypertension, a heart murmur, asthma, urinary incontinence, hypothyroidism, arthritis, neuropathy, fibromyalgia, and osteopenia. Her ocular history was notable for strabismus surgery performed at the age of five years. Aside from this, her ocular history was otherwise unremarkable. The patient reported no use of ocular medication. A review of systems, family history, and social history revealed no additional pertinent findings.

Upon comprehensive ophthalmic evaluation, her best-corrected visual acuity was 20/20 in both eyes (OU). The pupils were round and reactive to light, with no pupillary defect OU. Her refractive error was +2.00 sphere the right eye (OD) and +1.50, -0.25 x 88 OS, with an additional +1.25 OU. Intraocular pressure was measured using a Goldmann applanation tonometry with results of 13 mmHg OD and 17 mmHg OS. Her confrontation visual field test revealed full visual fields OU. Her sensory test showed a comitant alternating exotropia both at a distance and near. The ductions and versions were full. Her comprehensive slit-lamp examination revealed a giant translucent, fluid-filled conjunctival cyst observed temporally in the OS. Her dilated fundus examination showed no posterior segment pathology OU.

**Figure 1:**
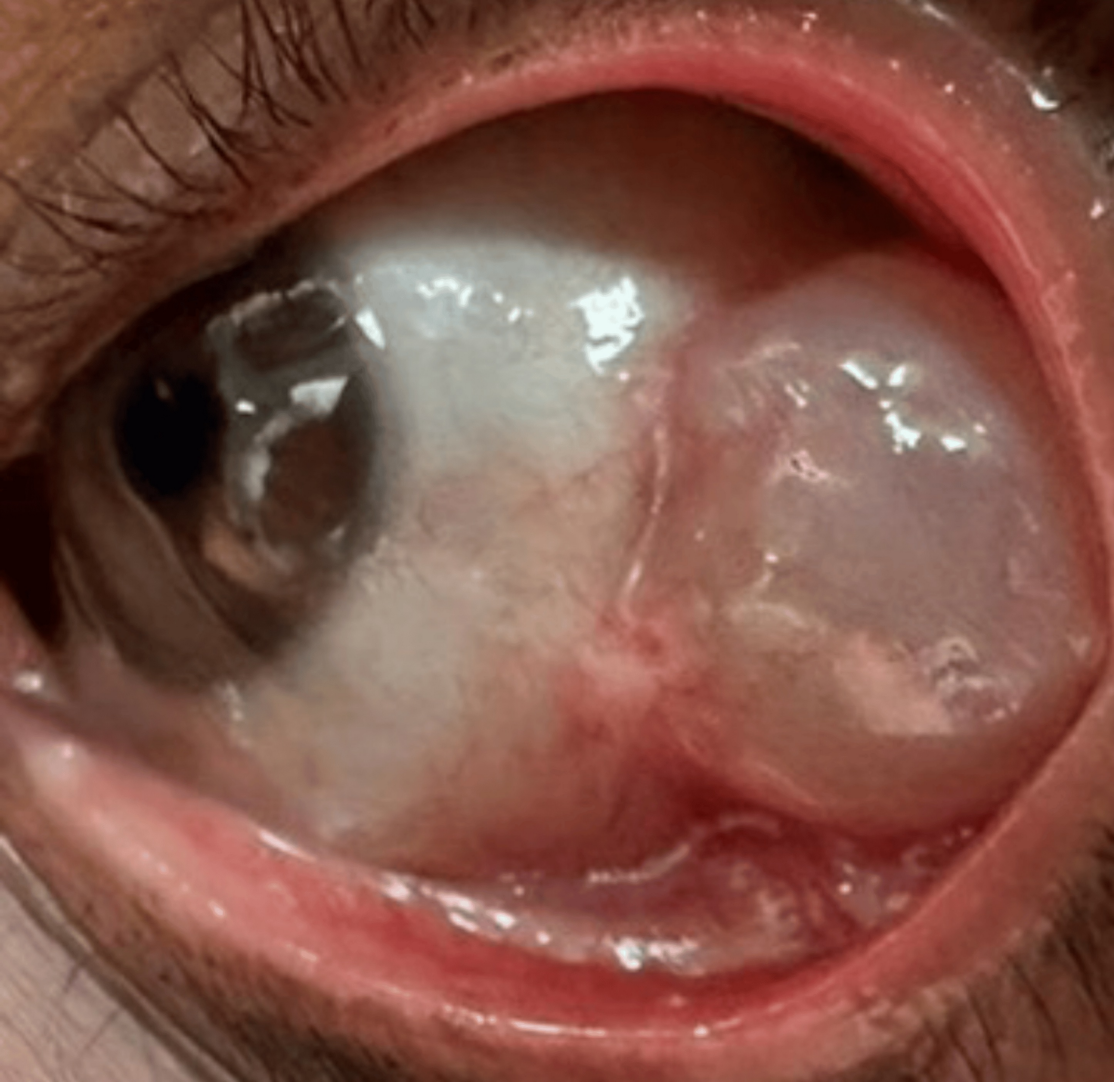
Left eye in adduction showing a giant conjunctival cyst in the temporal area

The patient was scheduled for an excision of the conjunctival cyst with conjunctival reconstruction. However, during the procedure, the cyst ruptured (during excision) and drained completely. Cauterization was performed on the tenons under the conjunctiva and on the area of the sclera in front of the muscle. One month later, the cyst (Figure 1) recurred, and subsequent computed tomography imaging of the orbits with contrast revealed a left orbital cyst lesion contiguous with and inseparable from the anterior aspect of the lateral rectus muscle, with no posterior orbital extension. The cyst lesion measured 1.3 cm anteroposteriorly, 0.6 cm transversely, and 1.4 mm craniocaudally.

The patient continued to experience headaches, as well as other kinds of pain and discomfort with eye movement, particularly when looking to the left. Her symptoms were significantly affecting her quality of life. To address these challenges, we opted for a treatment approach utilizing 20% TCA, prepared by aqueous dilution of 100% trichloroacetic acid (6.1 N TCA, Sigma-Aldrich, St. Louis, MO). The procedure was performed in the operating room under general anesthesia. To ensure corneal safety, the treatment was carefully targeted to the area of the cyst farthest from the cornea. The procedure began with complete aspiration of the cyst contents using a 27G needle attached to a 5 mL syringe containing 1 mL of 20% TCA. Without removing the syringe, the TCA was allowed to mix with the cyst fluid, further diluting the solution. The resulting mixture was then re-injected into the cyst cavity until the cyst reformed and turned white. The cyst was then re-aspirated until it became flat, and the needle was removed. The cyst cavity was thoroughly irrigated with balanced salt solution (BSS) using a cannula and compressed to ensure the complete removal of any residual acid. Throughout the procedure, profuse irrigation with BSS and the placement of a microsponge were employed to protect the cornea from any potential exposure to TCA.

**Figure 2:**
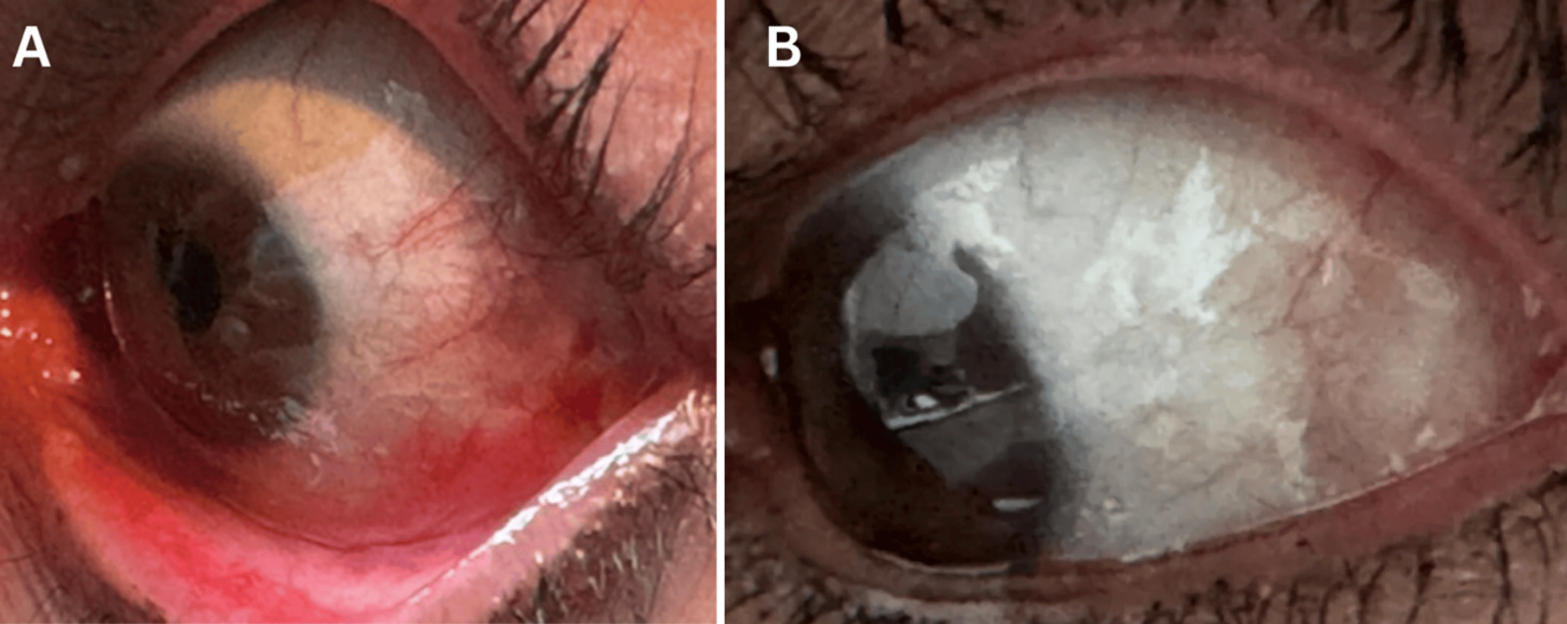
(A) Left eye in adduction in the operating room after the procedure; (B) Left eye in adduction eight months post procedure

Upon completion of the procedure, no corneal damage was observed. Only mild conjunctival abrasion was noted in the surrounding tissue (Figure 2A), which resolved completely within 24 hours. The patient was monitored over eight months post procedure, during which the cyst fully resolved without recurrence, leading to a significant improvement in symptoms, as shown in Figure 2B.

## Discussion

The conventional treatment for a conjunctival inclusion cyst is excision, although thermal cautery and yttrium-aluminum-garnet lasers have also been used as management for these cysts [6,7]. Alternative treatments for giant conjunctival inclusion cysts have been scarce since these cysts are prone to recurrence due to the complexity of the surgery and the potential for rupture. The patient described in this case underwent an initial surgical attempt to excise her cyst, but it was unsuccessful, resulting in recurrence.

This led us to explore alternative treatment options that could decrease the recurrence of this kind of cyst. In a previous case series, 10% TCA was used to treat the superficial conjunctival inclusion cysts, with no recurrence being reported [8]. Other case studies have also mentioned the use of 20% TCA to treat conjunctival epithelial inclusion cysts after evisceration [4,9] and post-strabismus surgery giant scleral cysts [10]. When discussing giant conjunctival cysts, Owji and Bolkheir [5] of the Shiraz University Medical School in Iran documented a similar approach in patients with idiopathic giant fornix cysts. The rationale behind this approach was that TCA, a chemical cauterizing agent, can effectively destroy cystic epithelial cells, thereby reducing the risk of recurrence [1,5]. However, the use of TCA has potential complications, mainly due to its caustic nature. One critical pitfall is the risk of corneal complications, such as corneal epithelial defects or even corneal scarring, which could lead to compromised vision if not managed appropriately. To mitigate these risks, close monitoring and protective measures, such as shielding the cornea with a microsponge and using extensive irrigation, are essential during the procedure to ensure that TCA does not contact the cornea or adjacent tissues.

There is no definitive treatment for the management of conjunctival cysts, and many of the proposed options have a high risk of recurrence. There is limited data on the management of giant conjunctival cysts after strabismus surgery and on the use of TCA in seeing eyes. In our study, we describe our approach to injecting TCA 20% in a giant conjunctival cyst, which we hope will serve as a foundation for further investigation.

## Conclusions

Recurrent giant conjunctival cysts present a challenge due to their potential to cause persistent discomfort, along with their tendency for rupture and recurrence following traditional surgical treatment. This case suggests that 20% TCA is a viable treatment option for recurrent giant conjunctival cysts, offering effective resolution with a reduced risk of complications. While our patient responded successfully to this treatment, prospective studies are warranted to further evaluate its long-term effectiveness and safety.
